# The Mirage of Upward Mobility: Conceptualization and Implications for Teen Dating Violence Prevention

**DOI:** 10.3390/children10111785

**Published:** 2023-11-06

**Authors:** Lídia Puigvert-Mallart, Susana León-Jiménez, Mar Joanpere, Mimar Ramis-Salas, Ramón Flecha

**Affiliations:** 1 Research and Department of Sociology, Faculty of Economics and Business, University of Barcelona, 08034 Barcelona, Spain; lidia.puigvert@ub.edu (L.P.-M.); susana.leon@ub.edu (S.L.-J.); mar.joanpere@ub.edu (M.J.); mimarramis@ub.edu (M.R.-S.); 2 Centre for Community, Gender and Social Justice, Institute of Criminology, Cambridge University, Cambridge CB3 9DA, UK; 3 Department of Education, Faculty of Educational Sciences and Psychology, University of Cordoba, 14071 Cordoba, Spain

**Keywords:** coercive discourse, attraction, peer pressure, status, risk factors, youth

## Abstract

Alcohol and drug abuse are recognized risk factors in scientific literature that can leave female adolescents vulnerable to experience teen dating violence (TDV) in their relationships. These risk factors are highlighted in prevention campaigns, empowering girls to proactively avoid or decline situations that may put them at risk of such violence. This study delves into an underexplored risk factor for TDV, the Mirage of Upward Mobility (MUM), and its connections to previously established elements: coercive discourse, peer pressure, and the pursuit of status. A total of 146 relevant studies on TDV, and factors related to it, have been reviewed. MUM is conceptualized as the erroneous belief that engaging in relationships with traditionally dominant and disrespectful partners increases social status and attractiveness, although in reality, what happens is that that status and that attractiveness decrease. The study discusses the existence and factors contributing to MUM and underscores its importance as a risk factor for experiencing TDV to include in prevention campaigns.

## 1. Introduction

Gender-based violence is an urgent public health concern that deeply worries academics, society, and public institutions worldwide [1,2]. Among the categories of gender violence, intimate partner violence stands out as the one with the highest prevalence rates [3]. It is recognized as a continuum of abuse, as opposed to the traditional standard approach as episodic incidents [4]. When such abusive behaviors occur among adolescents in relationships marked by power imbalances, control, manipulation, or harm, it is termed teen dating violence (TDV) [5]. According to the Centers for Disease Control and Prevention [6], TDV encompasses four types of behavior, physical violence, sexual violence, psychological aggression, and stalking, occurring both in person and through electronic means, such as social media websites and cellphones.

In the context of its impact on the future well-being and health of adolescents, addressing the issue of TDV emerges as a critical imperative for both research and practical efforts [7,8,9]. This is particularly vital because adolescents often connect their mental health with the quality of their relationships and social interactions [10]. The practices employed to combat and prevent TDV encompass recognizing risk factors and root causes, reshaping sociocultural norms, imparting education, providing training and information, and collaborating with justice systems [11]. Preventing TDV necessitates not only considering the already identified causes but also exploring and integrating new elements into the study.

Extensive research has been conducted to understand the risk factors associated with TDV among girls. Scientific evidence has established a clear link between excessive alcohol and/or drug consumption in girls and their vulnerability to abusive relationships [12,13,14,15,16,17,18], that mainly arises because this excessive consumption impairs their ability to identify and resist abusive behavior. These studies have also explored the roles of men and women in relation to alcohol abuse, leading to the development of prevention campaigns aimed at mitigating these risk factors.

However, it is crucial to continue analyzing the indicators already identified in studies and commonly reported by young people. In this pursuit, our comprehensive research review focuses on a phenomenon known as the “mirage of upward mobility” (MUM), prevalent in many of the relationships of youth and teens in relation to TDV [19,20]. It has largely been unexplored in the international scientific literature, with the exception of the competitive Framework Project R&D+I, Mirage of Upward Mobility and Socialization of Gender Violence, funded by the Spanish Ministry of Science and Innovation [21].

MUM, a concept introduced by Puigvert and Flecha [22], refers to a phenomenon where adolescents and young people mistakenly believe that being in a sexual-affective relationship with a disrespectful, sexist, or imposing partner will increase their attractiveness and social status. In reality, the opposite occurs, as their status and attractiveness often decrease. This misperception can lead them to engage in relationships associated with TDV [21].

Every man who engages in violence against women conforms to the norms of hegemonic masculinity [23]. These men adhere to a “bad boy” pattern, characterized by sexist, male chauvinist, and belligerent behavior [24]. The dominant traditional masculinity (DTM) role exercises power, belittling both women and men who do not follow violent and aggressive gender roles. Numerous studies have confirmed that boys conforming to the DTM role often engage in insults, humiliation, criticism, and the creation of a negative image of the girls they are involved with during and/or after the relationship [25,26,27,28]. This kind of boy can be perceived as attractive to girls due to the coercive discourse that associates appeal with such attitudes, influencing their choices [29,30,31].

While the research on MUM is primarily identified in heterosexual relations, it draws upon numerous life stories of girls to comprehend and identify the phenomenon for TDV prevention. This article, however, does not present fieldwork findings but aims to understand the constituent elements through a comprehensive literature review.

The purpose of this review is to shed light on the MUM phenomenon and its connection to TDV within adolescent relationships. In this article, we present the independent elements identified in literature that are linked to TDV and, when combined, contribute to the configuration of MUM as a risk factor for experiencing violence. We propose an analysis of these key elements to understand MUM, which includes the following: (a) the coercive discourse [29,30,32]; (b) peer group pressure [33,34,35,36]; and (c) the desire to enhance one’s status [37,38], specifically, the pressure to appear attractive and popular within one’s peer group. It is the combination of these three elements that contributes to the construction of MUM. By understanding it, the review aims to emphasize the need to address the MUM phenomenon as part of TDV prevention efforts.

## 2. Materials and Methods

This article is rooted in the findings of the Mirage of Upward Mobility and Socialization of Gender Violence competitive project, which was conducted between 2010 and 2012 and funded by the Ministry of Science and Innovation of Spain. The study encompassed several significant phases: (a) a literature review was conducted to understand the existing state of knowledge related to the processes of socialization associated with the phenomenon; and (b) empirical work was undertaken to refine the conceptualization of the phenomenon itself and its connection to TDV. This involved collecting 39 communicative life stories from adolescents, young women, and men hailing from Catalonia, Murcia, Euskadi, and Galicia (Spain). Additionally, 16 in-depth interviews were held with professionals who work in TDV prevention; (c) an in-depth analysis was performed on the measures and policies design for TDV prevention and mitigation; (d) the project also focused on developing guidelines for the prevention and mitigation of MUM. It emphasized the importance of the transversal dissemination of results, with an emphasis on knowledge transfer.

A total of 146 documents, comprising articles, books, grey literature (such as PhD research, reports, or official statistics), and press, have been analyzed (see Table S1). These documents encompass (a) article publications indexed in prominent databases like Web of Science, Sociological Abstracts, Eric, ERIH PLUS and Latindex, which hold the highest impact index in Social Sciences, sociology, and psychology; and (b) competitive research projects from both the European Research Framework Program (fifth, sixth, and seventh editions) and the Spanish R&D&I National Plan.

The keyword used in scientific literature searches included the following: violence, women and youth/romance, status and youth/gender violence, status and youth/gender violence and socialization/gender violence and love/gender violence and causes. The gathered contributions provided insights into the following: the socialization processes leading to TDV among young and adolescent populations; the dynamics of sexual-affective relationship choices, and elements related to the MUM phenomenon. This extensive compilation of information was then analyzed to identify connections between the factors contributing to TDV and those associated with MUM. The results are presented according to three key elements that contribute to MUM as a risk factor for TDV.

## 3. Results

### 3.1. Coercive Discourse: Attraction, Election and Violence

Understanding MUM necessitates delving into the dynamics of socialization in attractiveness and the intricate connections between attractiveness and violence. This connection can be traced to what has been termed the coercive discourse [30,32]. This discourse, expressed through language, effectively embodies peer pressure and serves as a pivotal precursor to gender-based violence. It characterizes communication within relationships marked by power imbalances, and shapes socialization in a manner that associates attractiveness with individuals who exhibit violent behavior, while deeming non-violent attitudes or relations as convenient but not-exciting and uninspiring [30,31]. Research indicates that this discourse can give rise to the cultivation of coercive preferences [32,39], exerting a significant influence on the choices girls make in their selection of sexual-affective relationships. As a result, MUM materialize when violence becomes an attractive quality, affecting sexual-affective relationships. Numerous authors have explored the social elements that underpin this phenomenon.

Literature addressing love and attractiveness underscores the social nature of the attraction [27,29,40]. Our preferences regarding who we find attractive and those we do not are influenced by social reasons. This includes our friend groups, family, school, leisure environments and mass media, in which frequently main characters are depicted as engaging in violent behavior. In these settings, it is not uncommon to encounter conversations and interactions that follow patterns of violence, fostering these models of attraction [41].

There exists a widespread form of socialization (though not exclusive nor equally internalized by everybody) that reinforces the connection between attraction and violence [27,39,40]. Certain roles, particularly those that are socially promoted as being more attractive, for example, masculine gender roles, are more problematic, conflictive, and violent—whether actual or potential. Researchers have even illuminated the existence of an attraction towards “conflictive” dynamics or aspects of “domination”. Their inquiries have explored the intricate links between power, social status, reputation, attractiveness, and desire. They have documented how some women express an attraction toward men who embody a masculine role characterized by power and domination over women [42,43,44,45,46].

The transition from childhood to adolescence introduces a maturity gap that can foster antisocial behavior among teenagers. During this phase, violent peers may be perceived as more attractive than well-behaved boys who were well valued during childhood [47]. A study conducted by Bukowski, Sippola, and Newcomb [48] suggests that the allure of aggressive roles increases after the transition to middle school, coupled with a heightened tolerance for violence.

Levi-Martin’s work [44] advances this research by developing fieldwork on the analysis of the perspective from both men and women, proving how power is an attractive element within the context of masculine roles. His study makes a critical distinction between the power exercised by one person over another and the power held as social status. He identifies that it is not merely social power (status) that leads some women to perceive certain masculine roles as more attractive, but the interpersonal power exerted over women. It is not merely a matter of occupying a position of power: rather, it involves establishing power dynamics among individuals. This author contends that both men and women perceive a masculine gender role that wields power over both genders as more attractive for certain reasons, though his study does not delve into what those specific reasons might be.

Rebellon and Manasse [49] conducted a study involving 1725 American adolescents aged 11 to 17, concluding that there exists a connection between delinquency and romantic involvement in adolescents. According to their findings, delinquent behavior seems to make young men more appealing to prospective partners due to the allure of their risk-taking tendencies. Similarly, Padrós [28] explores the attractiveness of masculine gender roles and posits that the most successful models have systematically fashioned themselves within a role of masculinity that occasionally includes aggression and violence.

Epstein et al. [50] conducted an analysis of adolescents’ perceptions of their peers in playground settings. Their findings reveal that girls tend to hold a more favorable view of boys who display tough behavior, as opposed to those who exhibit gentleness. Despite adolescents expressing a value for equal relations and rejecting violence and aggressiveness in their discourse, data suggest that an aggressive masculine role remains attractive within their romantic relationship, and their rejection of it is less pronounced than they declare [51,52,53,54].

Other researchers [37,55,56] focus on the links between the nature of lived relationship (hook-ups, romantic relationships, dating, etc.) and the development of TDV.

In light of this extensive backdrop, a central question emerges—the choice of a partner’s masculinity role [27,28,40,57,58]. This choice plays a crucial role in shaping MUM, as the phenomenon is not configured to the type of relationship but is intrinsically linked to the DTM role of the partner. In essence, only boys who embody a dominant masculine role [23,24,59,60,61,62,63,64,65,66] can drive the development of MUM.

When the socialization process fosters an attraction towards violence, it culminates in the selection of partners who embody a dominant traditional masculinity role marked by dominance and disdain, which is perceived as a “desirable” mate. It is then when MUM can appear. MUM is directly tied to the DTM role, and the selection of this partner for relationships is directly connected to the socialization of attraction toward domination and aggression. Subsequent sections will explore additional elements contributing to this socialization of attraction, the elements influencing the election of a DTM model, and, consequently, the potential to enter into MUM.

### 3.2. Peer Pressure: Interactions and First Relationships

The process of attraction is shaped through socialization, which encompasses various factors such as family, education, mass media, and, notably, peer groups [41,67,68,69]. Through their interactions and based on their observations in their surroundings, adolescents of both genders contemplate and envision their potential affective-sexual relations.

Adults play a significant role in this socialization process. According to McCarthy and Casey [70], the ideals of romantic relationships cherished by young boys and girls align with the expectations of adult men and women regarding desire, love, and bonding. Adolescents also describe similar emotions, thoughts, and behaviors associated with what adults perceive as “being in love”.

While Collins and colleagues [55] suggest that both family and peers have a comparable influence on decisions and actions related to their sexual-affective relationships, extensive research indicates that peer interactions play a more substantial role in establishing sexual-affective relations and susceptibility to suffer violence within them [71,72,73,74].

Specifically, the family, as a pivotal component of socialization [75,76], continues to wield significant influence over adolescents of both genders in various aspects of their lives, especially in decisions with long-term consequences, such as career choices and matters involving moral principles and ethical values. In contrast, peers exert more influence in shaping aspects related to youth culture, like style, preferences, and physical appearance [77].

The pressure exerted by peers to engage in sexual-affective relationships is the most significant form of interaction that triggers TDV [34,35,36]. Giordano [33] also highlights the substantial pressure experienced by adolescent girls from their peers to initiate sexual relationships with boys. This group pressure often prompts many young girls to engage in early stage sexual interactions, as observed by O’Sullivan and Meyer-Bahlburg [38].

Among the various types of peer group pressure, O’Sullivan and Meyer-Bahlburg [38] identify those targeting girls to participate in sexual games. In their research, it was found that every girl group between the ages of 9 and 11 reported engaging in games like “Spin the Bottle”, “Seven Minutes in Heaven”, “Run-Catch-Kiss” and “Manhunt”, which involve various forms of sexual contact among children of both genders. The pressure to participate in these games came from both boys and girls, with friends encouraging participation [38].

The establishment of sexual-affective relationships is also a critical element in the process of socialization. Smetana et al. [77] and Wiklund et al. [78] assert that the sexual-affective relationships formed during adolescence profoundly influence an individual’s psycho-social development, not only during this period but also in the subsequent stages of life. These relationships have far-reaching consequences and a significant impact on the socialization process of adolescents of both genders [33]. McCarthy and Casey [70] argue that each intimate relationship increases the likelihood of forming further relationships, affecting one’s future intimate relationships as an adult.

The evidence demonstrates that interactions within peer groups and the pressures exerted by these groups significantly influence the type of relationships chosen and the individuals selected for these relationships. Consequently, peer groups play a pivotal role in shaping MUM. On the one hand, they are a crucial element in influencing the attractiveness associated with masculinity models, as well as whether the promotion of the attraction to violence and aggression is encouraged or discouraged. On the other hand, peer groups can influence peers’ choices regarding the nature of relationships to establish (whether to engage in sexual activity, participate in sexual games, pursue casual hook-ups, or to establish romantic relationships). They can play a substantial role in either promoting or preventing MUM.

### 3.3. Status and Reputation: How I Want to Be Seen vs. How They See Me

Research [33,38] reveals that some girls believe they would attain greater social prestige and feel like adults, appearing more mature, by engaging sexual-affective relationships. These same authors concur with Collins and colleagues [55] when they assert that many adolescent girls experience pressure to initiate a sexual-affective relationship to gain acceptance and fit in with their peers. Some adolescent girls perceive that having a boyfriend not only makes them popular but also positions them as winners in the competition among their female friends. Thus, having a boyfriend for adolescent girls signifies maturity and popularity, particularly when the boyfriend is older and deemed attractive by their peers. Some girls even adopt a competitive feminine role, competing against each other to secure popularity among their peers. They might form clubs or groups to find the most attractive boy for them [38].

While girls are socialized with the idea that certain relationships would elevate their status [55], the reality demonstrates that female adolescents engaging in specific types of relations, such as sporadic and regular sexual encounters with different boys, are often subjected to criticism, insults, and disdain [79,80,81,82].

Girls who participate in certain “sexual games” or engage in sexual activity outside of stable relationships endure a downgrade in social image, a disparity not applied to boys participating in similar activities. The feminine role is depreciated, while the masculine role is appreciated [38]. These insights are pivotal for analyzing the socialization process that contributes to MUM. The pursuit of elevated status within a group leads some girls to form relationships with boys deemed attractive by their peers, and those adopting a DTM role are often the ones criticizing and belittling these girls.

These investigations highlight how girls face criticism for specific relationships—usually linked to sporadic relationships, participation in sexual games, and/or having multiple relationships—while boys do not receive a similar assessment. Hence, the necessity arises to explore the social interactions that contribute to this criticism and the nature of this criticism itself. This is an essential aspect of this study.

These investigations do not uncover the type of relationships that make girls feel uncomfortable or insecure about their status within the group. They do not delve into the attitudes of those who evoke these feelings of insecurity in girls or how this perceived lack of appreciation within the group can significantly influence their future relationship choices and self-perception. In MUM, the disdain towards girls is not rooted in the number or duration (stable or sporadic) of their relationships, but in the election of boys displaying disdainful and abusive behavior towards them.

At times, the type of relationship and/or the chosen individuals encouraged by peer groups are tied to the competitiveness emphasized by O’Sullivan and Meyer-Bahlburg [38], as well as the desire to present oneself as more mature or superior. In this context, MUM may be encouraged by the peer group, not only promoting attraction to violent models but also endorsing relationships with those adopting DTM as a symbol of being “better”. Therefore, the erroneous socialization of certain girls into believing that boys who hold them in contempt will elevate their status while, in reality, the opposite occurs as they are precisely the ones who decrease their status and generate bad reputation for them, which deserves further exploration.

It is also imperative to delve into what “bad reputation” and the degradation of a girl’s social image entail. What kinds of comments are made about these girls, and how do they affect their future relationships? Additionally, an analysis of girls’ perception of their social reputation at that moment and its connection to their current self-awareness is warranted.

A crucial aspect of MUM is the disparity in perception between girls who mistakenly believe that having a relationship with a boy adopting a DTM role elevates their status, and the reality where such relationships often diminish their standing among their peers.

## 4. Discussion and Future Directions

This review underscores the significance of MUM as a previously under-recognized risk factor for adolescents, directly linked to the development of TDV, and, more broadly, their overall well-being and mental health. This article defines the phenomenon and its elements, as well as its association with the sexual-affective relationships of some female adolescents.

Factors such as alcohol and drug abuse have been extensively examined in the scientific literature, confirming that the excessive consumption of these substances by women makes them more susceptible to abusive relationships. Prevention campaigns support these findings, but it is equally important to focus on the detection of MUM.

The present research highlights the strong connection between MUM and elements that have been well documented in the literature. Elements such as socialization in the attraction towards violence [28,40,43,44,48,49,58], coercive discourse [29,30,32,39] peer group pressure [77], the devaluation of social image, and the pursuit of status [33,37,38,82] are key elements linked to the onset of the MUM phenomenon.

Including this topic in prevention meetings is crucial as girls have the right to know about the existence of this phenomenon as a risk factor for experiencing TDV, working on its elements and presenting specific and real cases of MUM to be analyzed. The first key element that should be dealt with from a preventive viewpoint, is the fact that attraction is a social issue that develops through interactions based on the main socialization process and discourse that spreads the view of the DTM role as attractive [27,32,40,83]. The literature shows that it is necessary to clarify what is really improving social prestige and attractiveness and what is not, and to talk about the real consequences of having relationships with boys that follow a DTM role on the girls’ status and reputation. This need is even more urgent if we take into account the fact that the association between sexual-affective attraction to aggressive males and their attitudes and behaviors, as well as the peer interactions that force them into those disdainful relationships, have very negative effects on the health of victims, including suicidal ideation and suicide [39].

Overcoming MUM requires open dialogues within peer groups to discuss this issue. Likewise, separating attraction from violence needs to be addressed. Dialogues among peers will be more effective in achieving this than guidance provided by teachers or families. Encouraging dialogues that promote attraction and desire for alternative models of masculinity, such as egalitarian models, and discouraging the DTM role is crucial. However, for peers to engage in these interactions, they also need to understand all the elements that constitute MUM and how they contribute to the risk of TDV.

Evidence demonstrates the effectiveness of implementing prevention meetings on MUM. With the aim of fostering dialogues among peers and because of the cited research, workshops and opening dialogic spaces have been carried out to facilitate debates on MUM among adolescents. The Unitarian Platform against Gender Violence in Barcelona, Spain, in their annual forum, has organized events where secondary education students have the opportunity to participate in workshops and activities, including discussions on MUM. It has been observed that these dialogues make students aware of the existence of this phenomenon and encourage them to seek transformative strategies.

Whether in research, prevention, or intervention measures, it is essential to emphasize that the focus should not be on the quantity of relationships but on who individuals choose to have relationships with. This choice is what raises concerns about the consequences or risks of their relationships. Empowering girls to make informed and free choices regarding the kind of relationship they want to experience involves considering the evidence of the phenomenon and its components. Furthermore, understanding the factors leading to MUM and its consequences can inform future interventions and provide tools for systems to promote mental well-being among adolescents and healthier relationships.

Our study has some limitations that affect the review of the literature, as it primarily focuses on the impact of this phenomenon on girls engaged in heterosexual relationships. Future studies should explore whether MUM affects boys similarly or if there are differences in its elements when considering gender differences, as previous studies on risk factors for TDV have suggested [84]. Additionally, it would be valuable to investigate this phenomenon and its elements within the LGTBIQ+ population. This study represents a theoretical approach to the concept and the elements defining the MUM phenomenon. Future research should delve into case studies to understand how sexual-affective relationships resulting from MUM impact the mental health of adolescents and whether the loss of status and attractiveness associated with MUM negatively influences their well-being.

## Data Availability

No new data were created in this study. Data sharing is not applicable to this article.

## Supplementary Materials

The following supporting information can be downloaded at: https://www.mdpi.com/article/10.3390/children10111785/s1, Table S1: Systematization of the literature review.

## References

[1] Chacham A.S., Simão A.B.S., Caetano A.J. (2016). Gender-based violence and sexual and reproductive health among low-income youth in three Brazilian cities. Reprod. Health Matters.

[2] Mosavel M., Ahmed R., Simon C. (2011). Perceptions of gender-based violence among South African youth: Implications for health promotion interventions. Health Promot. Int..

[3] Stark L., Ager A. (2011). A Systematic Review of Prevalence Studies of Gender-Based Violence in Complex Emergencies. Trauma Violence Abus..

[4] Hickman L.J., Jaycox L.H., Aronoff J. (2004). Dating Violence among Adolescents. Trauma Violence Abus..

[5] Beatriz E.D., Lincoln A.K., Alder J., Daley N., Simmons F., Ibeh K., Figueroa C., Molnar B.E. (2018). Evaluation of a Teen Dating Violence Prevention Intervention among Urban Middle-School Youth Using Youth Participatory Action Research: Lessons Learned from Start Strong Boston. J. Fam. Violence.

[6] CDC Intimate Partner Violence Center for Disease Control and Prevention. https://www.cdc.gov/violenceprevention/intimatepartnerviolence/index.html.

[7] Silverman J.G., Mucci L.A., Hathaway J.E. (2001). Dating Violence Against Adolescent Girls and Associated Substance Use, Unhealthy Weight Control, Sexual Risk Behavior, Pregnancy, and Suicidality. JAMA.

[8] Lormand D.K., Markham C.M., Peskin M.F., Byrd T.L., Addy R.C., Baumler E., Tortolero S.R. (2013). Dating Violence Among Urban, Minority, Middle School Youth and Associated Sexual Risk Behaviors and Substance Use. J. Sch. Health.

[9] Exner-Cortens D. (2014). Theory and teen dating violence victimization: Considering adolescent development. Dev. Rev..

[10] Lukoševičiūtė-Barauskienė J., Žemaitaitytė M., Šūmakarienė V., Šmigelskas K. (2023). Adolescent Perception of Mental Health: It’s Not Only about Oneself, It’s about Others Too. Children.

[11] Asgary R., Emery E., Wong M. (2013). Systematic review of prevention and management strategies for the consequences of gender-based violence in refugee settings. Int. Health.

[12] Adams-Curtis L.E., Forbes G.B. (2004). College Women’s Experiences of Sexual Coercion. Trauma Violence Abus..

[13] Bouffard J.A., Miller H.A. (2014). The Role of Sexual Arousal and Overperception of Sexual Intent within the Decision to Engage in Sexual Coercion. J. Interpers. Violence.

[14] Calafat A., Hughes K., Blay N., Bellis M.A., Mendes F., Juan M., Lazarov P., Cibin B., Duch M.A. (2012). Sexual Harassment among Young Tourists Visiting Mediterranean Resorts. Arch. Sex. Behav..

[15] Cowley A.D. (2013). “Let’s Get Drunk and Have Sex.”: The complex relationship of alcohol, gender, and sexual victimization. J. Interpers. Violence.

[16] Girard A.L., Senn C.Y. (2008). The Role of the New “Date Rape Drugs” in Attributions About Date Rape. J. Interpers. Violence.

[17] Schnitzer S., Bellis M.A., Anderson Z., Hughes K., Calafat A., Juan M., Kokkevi A. (2009). Nightlife Violence: A Gender-Specific View on Risk Factors for Violence in Nightlife Settings: A Cross-Sectional Study in Nine European Countries. J. Interpers. Violence.

[18] Young A.M., McCabe S.E., Boyd C.J. (2007). Adolescents’ Sexual Inferences about Girls Who Consume Alcohol. Psychol. Women Q..

[19] Rué L.R., Martínez I., Flecha A., Álvarez P. (2014). Successful Communicative Focus Groups with Teenagers and Young People. Qual. Inq..

[20] Tellado I., López-Calvo L., Alonso-Olea M.J. (2014). Dialogic Design of Qualitative Data Collection for Researching the Mirage of Upward Mobility. Qual. Inq..

[21] Espejismo O.E. (2012). Espejismo Del Ascenso Y Socialización de la Violencia de Género.

[22] Alonso-Olea M.J., Mariño R., Rué L. (2012). The mirage of upward mobility in gender violence socialization. Rev. Interuniv. De Form. Del Profr..

[23] Connell R. (2012). Masculinity research and global change. Masc. Soc. Change.

[24] Flecha R., Puigvert L., Ríos O. (2013). The new masculinities and the overcoming of gender violence. Int. Multidiscip. J. Soc. Sci..

[25] Bird S.R. (1996). Welcome to the Men’s Club: Homosociality and the Maintenance of Hegemonic Masculinity. Gend. Soc..

[26] Currier D.M. (2013). Strategic Ambiguity: Protecting Emphasized Femininity and Hegemonic Masculinity in the Hookup Culture. Gend. Soc..

[27] Gómez J. (2015). Radical love: A Revolution for the 21st Century.

[28] Padrós M. (2012). Attractiveness male models in adolescence. Masc. Soc. Chang..

[29] Racionero-Plaza S., Ugalde-Lujambio L., Puigvert L., Aiello E. (2018). Reconstruction of Autobiographical Memories of Violent Sexual-Affective Relationships through Scientific Reading on Love: A Psycho-Educational Intervention to Prevent Gender Violence. Front. Psychol..

[30] Puigvert L., Gelsthorpe L., Soler-Gallart M., Flecha R. (2019). Girls’ perceptions of boys with violent attitudes and behaviours, and of sexual attraction. Palgrave Commun..

[31] Villarejo-Carballido B., Pulido C.M., Zubiri-Esnaola H., Oliver E. (2022). Young People’s Voices and Science for Overcoming Toxic Relationships Represented in Sex Education. Int. J. Environ. Res. Public Health.

[32] Puigvert L., Flecha R. (2018). Definitions of Coercive Discourse, Coerced Preferences and Coerced Hooking-Up. https://creativecommons.Org/licenses/by-nc-nd/4.0/.

[33] Giordano P.C. (2003). Relationships in Adolescence. Annu. Rev. Sociol..

[34] Abbey A., Parkhill M.R., Clinton-Sherrod A.M., Zawacki T. (2007). A Comparison of Men Who Committed Different Types of Sexual Assault in a Community Sample. J. Interpers. Violence.

[35] Luo B. (2008). Striving for comfort: “Positive” construction of dating cultures among second-generation Chinese American youths. J. Soc. Pers. Relatsh..

[36] Stern E., Cooper D., Greenbaum B. (2014). The Relationship Between Hegemonic Norms of Masculinity and Men’s Conceptualization of Sexually Coercive Acts by Women in South Africa. J. Interpers. Violence.

[37] Giordano P.C., Longmore M.A., Manning W.D. (2006). Gender and the Meanings of Adolescent Romantic Relationships: A Focus on Boys. Am. Sociol. Rev..

[38] O’Sullivan L.F., Meyer-Bahlburg H.F.L. (2003). African-American and Latina Inner-City Girls’ Reports of Romantic and Sexual Development. J. Soc. Pers. Relatsh..

[39] Puigvert L., Racionero-Plaza S., Lopez de Aguileta G., Tellado I., Molina S., Pulido-Rodríguez M.Á., Ugalde L., Flecha R. (2023). Disdainful hookups: A powerful social determinant of health. J. Urban Health.

[40] Gómez J. (2004). El Amor en la Sociedad del Riesgo.

[41] Sordé T., Aiello E., Castro M. (2017). Guía Para la Comunidad Educativa de Prevención y Apoyo a las Víctimas de Violencia Escolar.

[42] McDaniel A.K. (2005). Young Women’s Dating Behavior: Why/Why Not Date a Nice Guy?. Sex Roles.

[43] McKinnon C.A., Nelson C., Grossberg L. (1988). Desire and power: A feminist perspective. Marxism and the Interpretation of Culture.

[44] Levi-Martin J. (2005). Is power sexy?. Am. J. Sociol..

[45] Pyke K.D. (1996). Class-based masculinities. Gend. Soc..

[46] Ríos O., Christou M. (2010). Más allá del lenguaje sexista: Actos comunicativos en las relaciones afectivo-sexuales de los y las adolescentes. Revista Signos..

[47] Moffitt T.E. (1993). Adolescence-Limited and Life-Course-Persistent Antisocial Behavior: A Developmental Taxonomy. Psychol. Rev..

[48] Bukowski W.M., Sippola L.K., Newcomb A.F. (2000). Variations in patterns of attraction of same- and other-sex peers during early adolescence. Dev. Psychol..

[49] Rebellon C.J., Manasse M. (2004). Do “bad boys” really get the girls? Delinquency as a cause and consequence of dating behavior among adolescents. Justice Q..

[50] Epstein D., Kehily M., MacGhaill M., Redman P. (2001). Boys and Girls Come Out to Play. Men Masc..

[51] Amurrio M., Larrinaga A., Usategi E., Del Vella A. (2008). Violencia de Género en Las Relaciones de Pareja en Adolescentes Y Jóvenes de Bilbao. Conclusiones Finales.

[52] Eaton A.A., Rose S. (2011). Has Dating Become More Egalitarian? A 35 Year Review Using Sex Roles. Sex Roles.

[53] Urbaniak G.C., Kilmann P.R. (2003). Physical attractiveness and the “nice guy paradox”: Do nice guys really finish last?. Sex Roles.

[54] Urbaniak G.C., Kilmann P.R. (2006). Niceness and Dating Success: A Further Test of the Nice Guy Stereotype. Sex Roles.

[55] Collins W.A., Welsh D.P., Furman W. (2009). Adolescent romantic relationships. Annu. Rev. Psychol..

[56] Bearman Peter S., Moody J., Stovel K. (2004). Chains of Affection: The Structure of Adolescent Romantic and Sexual Networks. Am. J. Sociol..

[57] Duque E. (2006). Aprendiendo Para El Amor O Para la Violencia. Las Relaciones en Las Discotecas.

[58] Valls R., Puigvert L., Duque E. (2008). Gender Violence Among Teenagers. Violence Against Women.

[59] Carrigan T., Connell B., Lee J. (1985). Toward a new sociology of masculinity. Theory Soc..

[60] Castro M., Mara L.C. (2014). The social nature of attractiveness: How to shift attraction from the dominant traditional to alternative masculinities. Int. Multidiscip. J. Soc. Sci..

[61] Donaldson M. (1993). What is hegemonic masculinity?. Theory Soc..

[62] Hearn J. (2012). A Multi-Faceted Power Analysis of Men’s Violence to Known Women: From Hegemonic Masculinity to the Hegemony of Men. Sociol. Rev..

[63] Locke B.D., Mahalik J.R. (2005). Examining Masculinity Norms, Problem Drinking, and Athletic Involvement as Predictors of Sexual Aggression in College Men. J. Couns. Psychol..

[64] Quinn B.A. (2002). Sexual Harassment and Masculinity: The Power and Meaning of “Girl Watching”. Gend. Soc..

[65] Robinson K.H. (2005). Reinforcing hegemonic masculinities through sexual harassment: Issues of identity, power and popularity in secondary schools. Gend. Educ..

[66] Schrock D., Schwalbe M. (2009). Men, Masculinity, and Manhood Acts. Annu. Rev. Sociol..

[67] Berger P.L., Luckmann T. (1966). The Social Construction of Reality: A Treatise in the Sociology of Knowledge.

[68] West C., Zimmerman D.H. (1987). Doing Gender. Gend. Soc..

[69] Deutsch F.M. (2007). Undoing Gender. Gend. Soc..

[70] McCarthy B., Casey T. (2008). Love, Sex, and Crime: Adolescent Romantic Relationships and Offending. Am. Sociol. Rev..

[71] Adelman M., Kil S.H. (2007). Dating Conflicts: Rethinking dating violence and youth conflict. Violence Against Women.

[72] Antônio T., Koller S.H., Hokoda A. (2011). Peer Influences on the Dating Aggression Process Among Brazilian Street Youth. J. Interpers. Violence.

[73] Deming M.E., Covan E.K., Swan S.C., Billings D.L. (2013). Exploring Rape Myths, Gendered Norms, Group Processing, and the Social Context of Rape Among College Women. Violence Against Women.

[74] Keelan C.M., Schenk A.M., McNally M.R., Fremouw W.J. (2013). The Interpersonal Worlds of Bullies: Parents, peers and partners. J. Interpers. Violence.

[75] Akers A.Y., Yonas M., Burke J., Chang J.C. (2010). “Do You Want Somebody Treating Your Sister Like That?”: Qualitative Exploration of How African American Families Discuss and Promote Healthy Teen Dating Relationships. J. Interpers. Violence.

[76] Pilgrim N.A., Ahmed S., Gray R.H., Sekasanvu J., Lutalo T., Nalugoda F.K., Serwadda D., Wawer M.J. (2013). Sexual Coercion Among Adolescent Women in Rakai, Uganda. J. Interpers. Violence.

[77] Smetana J.G., Campione-Barr N., Metzger A. (2006). Adolescent Development in Interpersonal and Societal Contexts. Annu. Rev. Psychol..

[78] Wiklund M., Malmgren-Olsson E.B., Bengs C., Öhman A. (2010). “He Messed Me Up”: Swedish Adolescent Girls’ Experiences of Gender-Related Partner Violence and Its Consequences Over Time. Violence Against Women.

[79] Noonan R.K., Charles D. (2009). Developing Teen Dating Violence Prevention Strategies. Violence Against Women.

[80] Page E., Shute R., McLachlan A. (2014). A Self-Categorization Theory Perspective on Adolescent Boys’ Sexual Bullying of Girls. J. Interpers. Violence.

[81] Papp L.J., Hagerman C., Gnoleba M.A., Erchull M.J., Liss M., Miles-McLean H., Robertson C.M. (2015). Exploring Perceptions of Slut-Shaming on Facebook: Evidence for a Reverse Sexual Double Standard. Gend. Issues.

[82] Willis P. (1988). Learning to Labor: How Working Class Kids Get Working Class Jobs.

[83] Ruiz-Eugenio L., Racionero-Plaza S., Duque E., Puigvert L. (2020). Female university students’ preferences for different types of sexual relationships: Implications for gender-based violence prevention programs and policies. BMC Women’s Health.

[84] Sorrentino A., Santamato M., Aquino A. (2023). Individual, Familial, and School Risk Factors Affecting Teen Dating Violence in Early Adolescents: A Longitudinal Path Analysis Model. Societies.

