# Supplementary material for: The Mirage of Upward Mobility: Conceptualization and Implications for Teen Dating Violence Prevention
Source: Children (Basel). 2023 Nov 6;10(11):1785. doi: 10.3390/children10111785 (PMC10670360; doi:10.3390/children10111785)
Supplement: Supplementary file 1 [file children-10-01785-s001.zip › children-2667124-supplementary.pdf]

Supplementary Material

Table S1: Systematization of the literature review

Articles	Books	PhD thesis	Projects/Reports	Press	Statistics
Abbey, A., Parkhill, M. R., Clinton-Sherrod, A. M., & Zawacki, T. (2007). A comparison of men who committed different types of sexual assault in a community sample. *Journal of interpersonal violence, 22*(12), 1567-1580.			Alonso, M.J.., Beloki, N. & Gómez, A. (2010). Socialization to Prevent Gender Violence in the Basque Country. Unceta, A & Medrano, C. *Equality,Equity and Diversity: Educational Solutions in the Basque Country.* Reno: Centro for Basque Studies (University of Nevada)		
Adams-Curtis, L. E. & Forbes, G. B. (2004). College women's experiences of sexual coercion: a review of cultural, perpetrator, victim, and situational variables. *Trauma, violence, & abuse, 5* (2), 91 - 122.	Beck, U.; Beck-Gernsheim, E. (1998). *El normal caos del amo*r. Barcelona: El Roure.	*Melgar, P. (2009). Trenquem el silenci: superación de las relaciones afectivas y sexuales abusivas por parte de las mujeres víctimas de violencia de género., University of Barcelona. Barcelona.*	Antônio, T., Koller, S. H., & Hokoda, A. (2012). Peer influences on the dating aggression process among Brazilian street youth: A brief report. *Journal of interpersonal violence, 27*(8), 1579-1592	*Alandete, D. (2010, April 11). En manos de las Chicas Malas. El suicidio de Phoebe Prince, acosada por sus compañeros de instituto, conmociona a EE UU. El País*	CIS. (2004). *Barómetro de marzo. Estudio nº 2258. marzo 2004*. Madrid: Centro de Investigaciones Sociológicas.
Adelman, M. & Sea, H. (2007). Dating conflicts: rethinking dating violence and youth conflict. *Violence against women, 13*(12): 1296-1318.					
Akers, A. Y., Yonas, M., Burke, J., & Chang, J. C. (2011). "Do you want somebody treating your sister like that?" Qualitative exploration of how African American families discuss and promote healthy teen dating relationships. *Journal of interpersonal violence, 26*(11), 2165-2185.			Díaz-Aguado, M. J., & Carvajal, M. I. (2011). I*gualdad y prevención de la violencia de género en la adolescencia.* Madrid: Ministerio de Sanidad, Política Social Igualdad.		

Alonso-Olea, M.J.; Mariño, R. & Rue, L. (2012). El espejismo del ascenso en la socialización de la violencia de género [The mirage of upward mobility in gender violence socialization]. *Revista Interuniversitaria de formación del profesorado*, 73 (26,1): 75-88.

Asgary, R., Emery, E., & Wong, M. (2013). Systematic review of prevention and management strategies for the consequences of gender-based violence in refugee settings. *International health*, *5*(2), 85-91.

Azaola, E. (2009). Patrones, estereotipos y violencia de género en las escuelas de educación básica en méxico. *Revista De Estudios De Género. La Ventana, 7*, 45.

Bearman, P. S.; Moody J.; Stovel K. (2004). Chains of affection: the structure of adolescent romantic and sexual networks. *American Journal of Sociology*, Vol. 110, num. 1, 44-91.

Bearman, P. S.; Moody J. & Stovel K. (2004). Chains of affection: the structure of adolescent romantic and sexual networks. *American journal of sociology*, Vol. 110, num. 1, 44-91.

Beatriz, E. D., Lincoln, A. K., Alder, J., Daley, N., Simmons, F., Ibeh, K., Figueroa, C. & Molnar, B. E. (2018). Evaluation of a teen dating violence prevention intervention among urban middle-school youth using youth participatory action research: lessons learned

Berger, P., & Luckman, T. (1966). *La construcción social de la realidad*. Buenos Aires: Amorrotu.

CIS. (2004). *Barómetro de marzo. Estudio nº 2258. marzo 2004*. Madrid: Centro de Investigaciones Sociológicas.

Ríos, O. (2010). Socialització de gènere: la construcció de la masculinitat a l'escola. University of Barcelona. Barcelona

Amurrio, M., Larrinaga, A., Usategi, E., & Del Vella, A. (2010). V*iolencia de género en las relaciones de pareja de adolescentes y jóvenes de Bilbao. Conclusiones finales.* Ayuntamiento de Bilbao y Universidad del País Vasco.

El Mundo (2010, April 5). EEUU juzga a nueve adolescentes por acosar a una alumna que se suicidó.

Instituto de la Mujer (2010). *Estadísticas: Mujeres muertas por violencia de género a manos de su pareja o expareja.*Madrid: Instituto de la Mujer.

from Start Strong Boston. *Journal of family violence*, *33*(8), 563-578.

Bird, S. (1996). Welcome to the men's club: Homosociality and the maintenance of hegemonic masculinity. *Gender & society, 10*(2): 120-132.

Bouffard, J. A. & Miller, H. A. (2014). The role of sexual arousal and over-perception of sexual intent within the decision to engage in sexual coercion. *Journal of interpersonal violence, 29* (11), 1967-1986.

Browing, C. R., Leventhal, T., & Brooks-Gunn, J. (2005). Sexual initiation in early adolescence: The nexus of parental and community control. *American Sociological Review, 70*, 758-778.

Calafat, A., Hughes, K., Blay, N., Bellis, M. A., Mendes, F., Juan, M., Lazarov, P., Cibin, B. & Duch, M. A. (2013). Sexual harassment among young tourists visiting Mediterranean resorts. *Archives of sexual behavior*, *42*(4), 603–13.

Carrigan, T; Connell, B. & Lee, J. (1985). Toward a new sociology of masculinity. *Theory and society*, *14*(5), 551-604.

Castro, M., & Mara, L. C. (2014). The social nature of attractiveness: how to shift attraction from the dominant traditional to alternative masculinities. *International and multidisciplinary journal of social sciences, 3*(2), 182-206.

Duque, E. (2006). *Aprendiendo para el amor o para la violencia. las relaciones en las discotecas*. Barcelona: Hipatia.

EMAKUNDE, Instituto Vasco de la Mujer (2005). *La Coeducación en la Comunidad Autónoma Vasca. Informe*. Vitoria: Publicaciones del Gobierno Vasco.

Chacham, A. S., Simão, A. B. S., & Caetano, A. J. (2016). Gender-based violence and sexual and reproductive health among low-income youth in three Brazilian cities. *Reproductive health matters, 24*(47), 141-152.

Collins, W. A., Welsh, D. P., & Furman, W. (2009). Adolescent romantic relationships. *Annual Review of Psychology,* 631-652.

Connell, R. (2012). Masculinity research and global change. *Masculinities and social change, 1*(1), 4-18.

Cowley, A. (2014). Let's get drunk and have sex: the complex relationship of alcohol, gender, and sexual victimization. *Journal of interpersonal violence, 29* (7), 1258-1278

Currier, D. M. (2013). Strategic ambiguity: protecting emphasized femininity and hegemonic masculinity in the hookup culture. *Gender & society,* 27(5), 704-727

Deming, M. E. (2013). Exploring rape myths, gendered norms, group processing, and the social context of rape among college women: a qualitative analysis. *Violence against women, 19*(4):465-485.

Deutsch, F. (2007). Undoing gender. *Gender & society, 21* (1): 106-127.

Díaz-Aguado Jalón, M. J. (2005). La violencia entre iguales en la adolescencia y su prevención desde la escuela. *Psicothema* 2005. Vol. 17, no 4, 549-558.

Giddens, A. (1993). T*he transformation of intimacy: Sexuality, love and eroticism in modern societies*. Cambridge: Polity- Press.

Gómez, J. (2008). *El amor en la sociedad del riesgo: Una tentativa educativa (2nd ed.).* Barcelona: El Roure.

Gómez, J. (2015). *Radical love. A revolution for the 21st century.* Peter Lang: New York

Jaspard M. & équipe Enveff . (2003). *Les violences envers les femmes en france. une enquête nationale*. Paris: La documentation Française.

EMAKUNDE, Instituto Vasco de la Mujer (2009). *Violencia contra las mujeres. Informe.* Vitoria: Publicaciones del Gobierno Vasco.

Fundación Mujeres-UNED. (2003). *DETECTA. Estudio sobre el sexismo interiorizado, presente en el sistema de creencias de la juventud y adolescencia y su implicación en la prevención de la violencia de género en el contexto de pareja.* Madrid: UNED.

Díaz-Aguado, M. J. (2009). Prevenir la violencia de género desde la escuela. *Revista De Juventud, 66*, 31-46.

Donaldson, M. (1993). What is hegemonic masculinity? *Theory and society, 22*(5), 643-657.

Dunn, J. L. (1999). What love has to do with it: the cultural construction of emotion and sorority women's responses to forcible interaction. *Social problems, 46*(3), 440-459.

Eaton, A. A. & Rose, S. (2011). Has dating become more egalitarian? A 35 year review using. *Sex roles, 64*(11-12): 843-862.

Kimmel, M. (2000). *The gendered society.* New York: Oxford University Press.

MacKinnon, Catharine A. 1988. "Desire and Power: A Feminist Perspective." Pp. 105–21 in *Marxism and the Interpretation of Culture*, edited by Cary Nelson and Lawrence Grossberg. Urbana: University of Illinois Press.

Mead, G. H. (Ed.). (1990). E*spíritu, persona y sociedad: Desde el punto de vista del conductismo social*. Barcelona: Paidós.

Instituto de la Mujer. (2000). *La violencia contra las mujeres. Resultados de la macroencuesta*. Madrid: Instituto de la Mujer.

Instituto de la Mujer (2010). Estadísticas: Mujeres muertas por violencia de género a manos de su pareja o expareja.

Ministerio de Igualdad (2010). *Igualdad y Prevención de laViolencia de Género en la Adolescencia.*Estudio pres entado en julio de 2010. 0. Ministerio de Igualdad. Madrid.

Oliver, E. (2010-2012). *Espejismo del ascenso y socialización de la violencia de género* [The mirage of upward mobility and the socialization of gender violence] (RTD Project). Madrid: Ministry of Economy and Competitiveness, Spanish Government.

Padrós, M. (2007). *Models d'atracció dels i de les adolescents. Contribucions des de la socialització preventiva de la violència de gènere*. Generalitat de Catalunya: Institut Català de les Dones.

Rodríguez Menéndez, M. C. (2007). Opiniones de las maestras de Educación Infantil sobre el papel de la familia y la escuela en la configuración de la identidad de género. REIFOP, 10 (1).

Sordé, T., Aiello, E., & Castro, M. (2017). *Guía para la comunidad educativa de prevención y apoyo a las víctimas de violencia escolar*. Ministerio de Educación

Eisikovits, Z., Winstok, Z., & Fishman, G. (2004). The firts israeli national survey on domestic violence. *Violence Against Women, 10*(7), 729-748.

Elboj, C; Flecha, A. & Iñiguez, T. (2009). Modelos de elección y atracción de la población adolescente y su relación con la violencia de género: Propuesta para su prevención en base a los principios metodológicos de Comunidades de Aprendizaje. *Contextos Educativos, 12*, 95- 114

Epstein, D., Kehily, M., Mac an Ghaill, M., & Redman, P. (2001). Boys and girls come out to play: Making masculinities and feminities in school playgrounds. *Men and Masculinities, 4*(2), 158-172.

Exner-Cortens, D. (2014). Theory and teen dating violence victimization: Considering adolescent development. *Developmental Review*, *34*(2), 168-188.

Fernandez Rius, L. (2008). ¿Violencia invisible, o del éxtasis al dolor? *Estudos Feministas, Florianópolis, 16*(1): 288, 133-144

Flecha, R. (2009). Cambio, inclusión y calidad en las comunidades de aprendizaje. C&E. *Cultura y Educación. 21*(2), 157-170.

Flecha, R., Puigvert, L., & Ríos, O. (2013). The new masculinities and the overcoming of gender violence.

Oliver, E., & Valls, R. (2004). *Violencia de género. investigaciones sobre quiénes, por qué y cómo superarla*. Barcelona: El Roure.

Oliver, E., & Valls, R. (2004). *Violencia de género. investigaciones sobre quiénes, por qué y cómo superarla*. Barcelona: El Roure.

Straus, M., Gelles, R., & Steimetz, S. (1980). *Behind closed doors. Violence in the american family.* Nueva York: Doubleday.

Willis, P. (1988). *Aprendiendo a trabajar. Cómo los chicos de la clase obrera consiguen trabajos de la clase obrera*. Madrid: Ediciones Akal.

Alonso, M.J., Beloki, N. & Gómez, A. (2010). Socialization to Prevent Gender Violence in the Basque Country. In A. Unceta & C. Medrano. *Equality,Equity and Diversity: Educational Solutions in the Basque Country.* Center for Basque Studies, Reno: University of Nevada

Valls, R. (2004-2005). *Educació en valors per la prevenció de la violència de gènere als Instituts d'Educació Secundària*. Generalitat de Catalunya: Agència de Gestió d'Ajuts Universitaris i de Recerca.

Valls, R. (2008). *Violencia de género en las universidades españolas. Plan Nacional I+D*. Madrid: Instituto de la Mujer.

International and multidisciplinary journal of social sciences, 2(1), 88-113

Forbes, G. B., Adam-Curtis, L. E., & White, K. B. (2004). First- and second-generation measures of sexism, rape myths and related beliefs, and hostility toward women. *Violence Against Women, 10*, 236-261

Geldschläger, H., Ginés, O., Ponce, A. (2009). Jóvenes en la intervención para hombres que ejercen violencia de género: dificultades y propuestas. *Revista de juventud, 66,* 197-215,

Giordano, P. C. (2003). Relationsehips in adolescence. *Annual Review of Sociology, 29*, 257-281.

Giordano, P. C., Longmore, M. A., & Manning, W. D. (2006). Gender and the meanings of adolescent romantic relationships: A focus on boys. *American Sociological Review, 71*, 260-287.

Girard, A. L. & Senn, C. Y. (2008). The role of the new "date rape drugs" in attributions about date rape. *Trauma, violence, & abuse, 23*(1), 3 - 20. doi: 10.1177/0886260507307648

Flecha, A. & Puigvert, L. (2010). Contributions to social theory from dialogic feminism: giving voice to all women. In Chapman, D. (ed.). *Examining Social Theory*. New York: Peter Lang.

Willis, P. (1988). *Aprendiendo a trabajar. Cómo los chicos de la clase obrera consiguen trabajos de clase obrera. [Learning to labor: how working class kids get working class jobs]*. Madrid: Akal

Gómez, A; Puigvert, L. & Flecha, R. (2011). Critical communicative methodology: informing real social transformation through research. *Qualitative Inquiry, 17,* (3) 235-245

Hamilton, L. & Armstrong, E. (2009). Gendered sexuality in young adulthood. Double binds and flawed options. *Gender & society,* 23 (5): 589-616.

Hearn, J. (2012). A multi-faceted power analysis of men's violence to known women: from hegemonic masculinity to the hegemony of men. *The sociological review, 60*, 589-610

Hickman, L. J., Jaycox, L. H., & Aronoff, J. (2004). Dating violence among adolescents: Prevalence, gender distribution, and prevention program effectiveness. *Trauma, violence, & abuse*, *5*(2), 123-142.

Levi Martin, J. (2005). Is power sexy? *American Journal of Sociology,* vol 111, Num. 2, 408-446.

Livingston, J., Buddie, A., & VanZile-Tamsen, M.T and C. (2004) The role of sexal precedence in verbal sexual coercion. *Psychology of Women Quarterly, 28*, 287–297.

Lomas, C. (2007). ¿La escuela es un infierno? Violencia escolar y construcción cultural de la masculinidad. *Revista de educación, 342*, 83-101

Lukoševičiūtė-Barauskienė, J., Žemaitaitytė, M., Šūmakarienė, V., & Šmigelskas, K. (2023). Adolescent

Perception of Mental Health: It's Not Only about Oneself, It's about Others Too. Children, 10(7), 1109.
McCarthy, B.; Casey T. (2008). Love, Sex, and crime: adolescent romantic relationships and offending. *American Sociological Review*, 73; 944-969.
Oliver, E; Soler, M & Flecha, R. (2009) Opening schools to all (women): efforts to overcome gender violence in Spain. *British Journal of Sociology of Education, 30*(2), 207 -218.
Padrós, M. (2012). Modelos de atractivo masculinos en la adolescencia. [Attractiveness male models in adolescence]. *Masculinities and social change, 1*(2): 165-183.
Padrós, M., Aubert, A. & Melgar, P. (2010). Modelos de atracción de los y las adolescentes. Contribuciones desde la socialización preventiva de la violencia de género [Models of attraction for adolescents. Contributions from the preventive socialization of gender violence]. *Pedagogía Social. Revista Interuniversitaria, 17*, 73-82
Page, E., Shute, R., & McLachlan, A. (2015). A self-categorization theory perspective on adolescent boys' sexual bullying of girls. *Journal of interpersonal violence*, *30*(3), 371-383.

Papp, L. J. (2015). Exploring perceptions of slut-shaming on Facebook: evidence for a reserve sexual double standard. *Sex Roles, 32*(1), 57-76

Pilgrim, N. A. (2013). Sexual coercion among adolescent women in Rakai, Uganda: Does family structure matter? *Journal of interpersonal violence, 28*(6): 1289-1313

Puigvert, L. (2014). Preventive socialization of gender violence. *Qualitative inquiry, 20* (7): 839-843

Puigvert, L., & Flecha, R. (2018). Definitions of Coercive Discourse, Coerced Preferences and Coerced Hooking-up. This Work is Licensed Under the Creative Commons Attribution- NonCommercial-NoDerivatives 4.0 International License.

Puigvert, L., Gelsthorpe, L., Soler-Gallart, M., & Flecha, R. (2019). Girls' perceptions of boys with violent attitudes and behaviours, and of sexual attraction. *Palgrave Communications*, *5*(1), 56.

Puigvert, L., Racionero-Plaza, S., Lopez de Aguileta, G., Tellado, I., Molina, S., Pulido-Rodríguez, M. Á.,

Ugalde, L., & Flecha, R. (2023). Disdainful hookups: a powerful social determinant of health. Journal of urban health, 1-8.

Pyke, K. (1996). Class-based masculinities: the interdependence of gender, class, and interpersonal power. *Gender & society,* 10 (5): 527-549

Quinn, B. A. (2002). Sexual harassment and masculinity. The power and meaning of «girl watching». *Gender & society, 16*(3), 386-402.

Racionero-Plaza, S.; Ugalde-Lujambio, L.; Puigvert, L. & Aiello, E. (2018). Reconstruction of Autobiographical Memories of Violent Sexual-Affective Relationships Through Scientific Reading on Love: A Psycho-Educational Intervention to Prevent Gender Violence. *Frontiers in psychology*, *9*.

Rebellon, C. J., & Manasse, M. (2004). Do "Bad boys" really get the girls? deliquency as a cause and consequence of dating behavior among adolescents. *Justice Quarterly, 21*(2), 355-389.

Ríos, O. & Christou, M. (2010). Más allá del lenguaje sexista. Actos comunicativos en las relaciones afectivo-sexuales de los y las adolescentes. *Revista Signos, 43*(2), 311-326.

Robinson, K. H. (2005). Reinforcing hegemonic masculinities through sexual harassment: issues of identity, power and popularity in secondary schools. *Gender and Education, 17*(1), 19-37.

Rodríguez Menéndez, M. C. (2007). Opiniones de las maestras de Educación Infantil sobre el papel de la familia y la escuela en la configuración de la identidad de género. Revista Electrónica Interuniversitaria de Formación del Profesorado, 10(1), 1-9

Rué, L. R.; Martínez, I.; Flecha, A. & Álvarez, A. (2014). Successful communicative focus groups with teenagers and young people. How to identify the mirage of upward mobility, *Qualitative inquiry 20*(7), p. 863-869.

Ruiz, J., Expósito, F., & Bonache, H. (2010). Adolescent witnesses in cases of teen dating violence: An analysis of peer responses. *European Journal of Psychology Applied to Legal Context, 2*(1).

Ruiz-Eugenio, L., Racionero-Plaza, S., Duque, E., & Puigvert, L. (2020). Female university students' preferences for different types of sexual relationships: implications for gender-based violence prevention programs and policies. *BMC women's health*, *20*(1), 1-13.

Schalet, A. (2010). Sexual Subjectivity Revisited: The significance of Relationships in Dutch and American Girls' Experiences of Sexuality. *Gender & society, 24* (3): 304-329

Schnitzer, S.; Bellis, M.A.; Anderson, Z.; Hughes, K.; Calafat, A.; Juan, M.; & Kokkevi, A. (2010). Nightlife violence: a gender-specific view on risk factors for violence in nightlife settings: a cross-sectional study in nine European countries. *Journal of interpersonal violence, 25* (6), 1094-1112.

Schrock, D.; & Schwalbe, M. (2009). Men, Masculinity, and Manhood Acts. *Annual Review of Sociology, 35*, 277-295

Silverman, J. G., Raj, A., Mucci, L. A., & Hathaway, J. E. (2001). Dating violence against adolescent girls and associated substance use, unhealthy weight control, sexual risk behavior, pregnancy, and suicidality. *Jama*, *286*(5), 572-579.

Smetana, J. G., Campione-Barr, N., & Metzger, A. (2006). Adolescent development in interpersonal and societal contexts. *Annual Review of Psychology, 57*, 255-284.

Ubillos, S; Zubieta,E; Páez, D; Deschamps, J.C; Ezeiza, A y Vera,A. (2001) Amor, Cultura y Sexo. *Revista electrónica de Motivación y Emoción, 4*, 8-9

Stark, L., & Ager, A. (2011). A systematic review of prevalence studies of gender-based violence in complex emergencies. *Trauma, Violence, & Abuse*, *12*(3), 127-134.

Stern, E., Cooper, D. & Greenbaum, B. (2015). The relationship between hegemonic norms of masculinity and men's conceptualization of sexually coercive acts by women in South Africa. *Journal of interpersonal violence, 30*(5), 796-817.

Tellado, I., López-Calvo, L., & Alonso-Olea, M. J. (2014). Dialogic design of qualitative data collection for researching the mirage of upward mobility. *Qualitative inquiry*, *20*(7), 856-862.

Urbaniak, G. C. & Kilmann, P. R. (2003). Physical attractiveness and the "nice guy paradox": Do nice guys really finish last? *Sex roles, 49*(9-10), 413-426

Urbaniak, G. C. & Kilmann, P. R. (2006). Niceness and dating success: A further test of the nice guy stereotype. *Sex roles, 55*(3-4), 209-224

Valls, R.; Puigvert, L. & Duque, E. (2008). Gender violence among teenagers. Socialization and prevention. *Violence against women. 14*(7), 759-785.

Vidu, A., Schubert, T., Muñoz, B, & Duque, E. (2014). What students say about gender violence within universities: Rising voices from the communicative methodology of research. *Qualitative inquiry, 20* (7), 883-888

Wahn, M. H., Culbreth, R., Salazar, L., Masyn, K., & Kasirye, R. (2016). Typologies of the gender-based

violence, hiv, and alcohol use syndemic among youth in Kampala. *Injury prevention, 22*(2).

West, C. & Zimmerman, D. (1987). Doing gender. *Sex roles, 1*(2), 125-151

Wiklund, M., Malmgren-Olsson, E. B., Bengs, C. & Öhman, A. (2010). “He messed me up”: Swedish adolescent girls’ experiences of gender-related partner violence and its consequences over time. *Violence against women*, *16*(2), 207-232.

Young, A. M., McCabe, S. E., & Boyd, C. J. (2007). Adolescents' sexual inferences about girls who consume alcohol. *Psychology of women quarterly, 31*(3), 229-240.
